# Prevalence of acute diarrhea and water, sanitation, and hygiene (WASH) associated factors among children under five in Woldia Town, Amhara Region, northeastern Ethiopia

**DOI:** 10.1186/s12887-021-02668-2

**Published:** 2021-05-11

**Authors:** Weldehawariyat Getahun, Metadel Adane

**Affiliations:** 1 Organization for Rehabilitation and Development in Amhara (ORDA) Ethiopia, Bahir Dar, Ethiopia; 2 Department of Environmental Health, College of Medicine and Health Science, Wollo University, Dessie, Ethiopia

**Keywords:** Acute diarrhea, Prevalence, Hygiene, Sanitation, Water supply, Under‐five children

## Abstract

**Background:**

Diarrhea among children under five is one of the significant public health concerns in developing countries, such as Ethiopia that is mainly attributed to inadequate water, sanitation and hygiene (WASH) services. Lack of data on the prevalence and factors associated with acute diarrhea in Woldia Town impedes the effectiveness of WASH programs in the area. Therefore, the aim of this study was to investigate the prevalence and WASH-associated factors of acute diarrhea among under-five children in this area. This study will help guide local diarrhea prevention and control programs.

**Methods:**

A community-based cross-sectional study was conducted among 485 children under age five from March to June 2018. The study participants were allocated proportionally and then households with children of this age group were selected from each *kebele* (the smallest administrative unit in Ethiopia) using a systematic random sampling technique. Data were collected from mothers/caregivers of the under-five children using a structured questionnaire and on-the-spot observation checklist. A binary logistic regression model with 95 % CI (confidence interval) was used to measure the association between dependent and independent variables. From the multivariable analysis, variables with a *p*-value < 0.05 were taken as factors significantly associated with acute diarrhea among under-five children.

**Results:**

The prevalence of acute diarrhea among the children was 17.6 % (95 % CI: 14.2–21.0 %). Almost two-thirds 307 (63.4 %) of study participants' main source of drinking water was a private tap; 320 (66.1 %) of households used less than 20 l per capita per day. About one-fifth 99 (20.5 %) of households used an improved sanitation facility. Only one-fifth (21.1 %) of the latrines had nearby handwashing facilities. Less than half 225 (46.5 %) of mothers/caregivers had good handwashing practice at critical times. Water consumption of less than 20 l per capita per day (adjusted odds ratio [AOR] = 2.45; 95 % CI: 1.36–5.84), unimproved sanitation facility (AOR = 3.57; 95 %CI: 1.64–6.51), practicing unsafe child feces disposal (AOR = 2.51; 95 % CI: 1.69–4.64), poor handwashing practice at critical times (AOR = 1.85; 95 % CI: 1.34–3.56) and having no information about diarrhea being prevented by handwashing with water and soap (AOR = 3.12; 95 % CI: 1.64–6.27**)** were significantly associated with acute diarrhea.

**Conclusions:**

More than one in six children under age five had acute diarrhea, a proportion that could be considered relatively high. We recommend that government organizations and concerned stakeholders strengthen urban WASH programs to focus on increasing the availability of sufficient water for adequate daily consumption, and promote safe disposal of child feces and good handwashing practices at critical times. Further effort is needed to sensitize mothers/caregivers about diarrhea prevention through effective WASH activity to reduce the burden of this problem among children under five.

## Introduction

During the past two centuries, the proportion of the world’s population living in cities and towns has grown from about 5 % to more than 50 %. The process of rapid urbanization, which started in Europe and North America after the Industrial Revolution in the late 18th century, has occurred in both developed and developing countries, resulting in the growth of many large urban areas [1, 2]. Because of rapid urbanization in developing countries, diarrhea among under-five children is now a major public health problem and the second leading cause of death in urban areas [3], according to a 2009 report by the World Health Organization (WHO) and United Nations International Children’s Emergency Fund (UNICEF). Globally, nearly one in five child deaths or about 1.5 million each year are due to diarrhea which is more than AIDS, malaria and measles combined [4].

Worldwide, about eight million children died in 2010, mainly due to poor sanitation facilities and unhygienic conditions [5]. An estimated 88 % of diarrheal deaths worldwide were attributed to unsafe water, inadequate sanitation, and poor hygiene practices [6]. Based on a World Gastroenterology Organization 2012 report, globally 78.0 % of all cases of diarrhea among children and adults occur in Africa and Southeast Asia [7]. According to a 2014 WHO report for low- and middle-income countries, issues of inadequate water, sanitation, and hygiene (WASH) account for 361,000 deaths in a year or over 1,000 deaths per day [8].

Diarrhea is common in the developing world due to the prevalence of unsafe drinking water, inadequate sanitation facilities, poor hygiene practices, a lack of household water treatment and unsafe water storage [9]. Improvements in access to safe water and adequate sanitation, along with the promotion of good hygiene practices (especially handwashing), greater availability and use of oral rehydration salts, rotavirus vaccination, increased coverage of measles immunizations, and exclusive breastfeeding until the age of 6 months can help prevent acute diarrhea in children under five [6, 7]. Each child under five in resource-limited countries in these regions, including Ethiopia, experiences an average of three annual episodes of acute diarrhea with the direct consequences of faltering growth, malnutrition, and impaired cognitive development [10].

Studies across Ethiopia have determined the prevalence of diarrhea among under-five children, including in Wolita Soddo Town (11 %) [11], Sidama Zone (13.6 %) [12], Hawassa Town (14.0 %) [13], Debre Berhan Town (16.4 %) [14], Sheka Zone (21.8 %) [15], North Gondar Zone (22.1 %) [16], Benishangul Gumuz Regional State (22.1 %) [17], Arba-Minch District (30.5 %) [18], Enderta Wereda (35.6 %) [19] and in Nekemte Town (28.9 % [20]. These studies revealed various factors related to acute diarrhea. A study in urban areas of Ethiopia revealed that inadequate sanitation facilities [21], interrupted water supply [22] and poor handwashing practice [23] were WASH-associated factors related to acute diarrhea among under-five children.

Although Ethiopia has been implementing an urban WASH program [24–26] and urban health extension program [27], diarrhea is still a major public health concern. This might be due to the fact that improving children’s health in urban areas is a complicated problem, as mentioned in other studies [28, 29], requiring updated evidence for targeted interventions. The 2016 annual report of the Woldia Town Health Office identified diarrhea as one of the top ten causes of morbidity and mortality among children under five. However, lack of local information on the prevalence and WASH-associated determinants of diarrhea in this age group is a challenge that impedes the effectiveness of the area’s WASH programs to control diarrhea.

Therefore, the aim of this study was to determine the prevalence of acute diarrhea and WASH-associated factors among children under five in Woldia Town. The findings of this study will contribute to the prevention of diarrhea by strengthening the local urban WASH program. Furthermore, the findings may also contribute to the efforts to monitor progress towards the achievement of the UN Sustainable Development Goals (SDGs) of 2030, to end preventable deaths of newborns and children under five years of age [30].

## Methods

### Study design and study area description

A community-based cross-sectional study was conducted among children under five in Woldia Town from March to June 2018. Woldia Town is the main town of North Wollo Zone, in Amhara Region, 520 km north of Addis Ababa. Woldia has a total population of 71,460, of which 35,873 are male and 35,587 female [31]. According to data provided by Woldia Health Office, there were a total of 9,676 children under age five in 7,921 local households. The town has a total of 10 *kebeles* (*Kebele* is the smallest administrative unit in Ethiopia, each consisting of a population of about 5,000).

### Source and study populations

The source population for this study consisted of all children under age five living in Woldia Town before the commencement of the study’s data collection. The study population consisted of systematically selected children under five from all 10 *kebeles* in Woldia Town. Eligible children with bloody and/or persistent diarrhea two weeks prior to the survey were excluded.

### Sample size determination and sampling technique

The sample size for this study was calculated using a single population proportion formula [*Z*_1_-_푎/2_]^2 *^P[1-*P*]/*d*^2^ [32]. The following assumptions were considered: 12.2 % prevalence (*P*) of acute diarrhea among children under five was taken from 2016 Ethiopia Demographic and Health Survey (EDHS) [33], 3 % margin of error (*d*), *Z*_1_-_푎/2_ at 95 % CI (confidence interval) = 1.96, and since the source population of 9,676 children under five in Woldia Town was less than 10,000, the following sample size correction formula was used: We considered a 10 % non-response rate to compensate for probable reduction in sample size caused by refusal of some to participate or failure to complete the study. Then the final sample size was 485.

Of the total 7,921 households with children under five, 787 households were registered in *Kebele* One, 949 in *Kebele* Two, 720 in *Kebele* Three, 1,120 in *Kebele* Four, 870 in *Kebel*e Five, 741 in *Kebele* Six, 781 in *Kebele* Seven, 945 in *Kebele* Eight, 511 in *Kebele* Nine, and 497 in *Kebele* Ten. In each *kebele* the number of households with children under five, house numbers, and other unique labels were provided by health extension workers.

Households with children under five were selected from each *kebele* and study participants were allocated proportionally. A sampling frame was made within households that had at least one child under five. Households with children under five were selected using a systematic sampling technique. To identify the first eligible household that included a child of this age group in each *kebele*, random sampling was used. From households with two or more children under five, one child was selected at random.

### Operational definitions

#### Acute diarrhea

Acute diarrhea was denoted as yes (1), indicating the presence of acute diarrhea, or no (0), indicating the absence of acute diarrhea during the two weeks prior to the survey; and was identified by asking the participants’ mothers/caregivers questions based on WHO-defined signs and symptoms of diarrhea of the child having abnormal loose or watery stools three or more times a day [34]. We adopted a two-week recall period as specified in the World Gastroenterology Organization global guidelines for acute diarrhea surveys [7].

#### Improved sanitation facility

The study used WHO/UNICEF Joint Monitoring Program (JMP) for Water Supply and Sanitation definition of improved facilities as those that ensure hygienic separation of human excreta from human contact and include flush or pour-flush to a piped sewer system, septic tank, pit latrine, ventilated improved pit latrine, pit latrine with slab and composting toilet [35].

#### Unimproved sanitation facility

The study used WHO/UNICEF JMP for Water Supply and Sanitation of WHO and UNICEF definition of unimproved facilities as those that do not ensure hygienic separation of human excreta from human contact including flush or pour-flush to other than a piped sewer system, pit latrine without slab or open pit, bucket, hanging toilet or hanging latrine, no facilities or bush or field or shared toilet [35].

#### Safe child feces disposal

This study used WHO/UNICEF JMP for Water Supply and Sanitation definition of safe child feces disposal as child defecation into a latrine and/or disposal of child’s stool into a latrine [36].

#### Unsafe child feces disposal

This study used the WHO/UNICEF JMP for Water Supply and Sanitation definition of unsafe child feces disposal as child feces put/rinsed into a drain or ditch, thrown into garbage, left in the open field, or buried [36].

#### Proper solid waste disposal

Disposal of solid waste at a legally authorized place [37].

#### Improper solid waste disposal

Disposal of solid waste at a legally unauthorized place [37].

#### Proper liquid waste discharge

Discharge of liquid waste through mesh wire and municipal liquid discharge methods.

#### Improper liquid waste discharge

Discharge of liquid waste into an open ditch outside/inside the house compound.

#### Critical times for handwashing practice

Handwashing with water and soap at five critical times: before preparing food, before feeding a child, before eating, after defecation and after cleaning a child who has defecated [38].

#### Good handwashing practice at critical times

Mothers/caregivers who reported washing their hands using water and soap at three or more of the five critical times during the two weeks prior to the survey.

#### Poor handwashing practice at critical times

Mothers/caregivers who reported washing their hands using water and soap at two or fewer of the five critical times or who had not washed at all during the two weeks prior to the survey.

### Study variables

The outcome variable was presence of acute diarrhea (yes/no) in children under age five in the two weeks prior to the survey. The independent variables were socioeconomic/demographic and WASH-related factors. Socioeconomic/demographic variables that were self-reported by the study participants were mother’s/caregiver’s education level, occupation, age (years), religion, marital status, ethnicity, number of children under five in the household, child’s age, child’s sex, birth order of child, child’s father’s education and occupation status, house ownership, household size and household economic status (wealth status). Wealth status of the study participant household was estimated using principal component analysis (PCA).

Of the WASH-related factors, water-related variables that were self-reported were: type of water source, time needed for household members to obtain drinking water, water consumption per capita per day, interruption of water supply in the previous two weeks, household water storage in the past two weeks, types of container used for water storage, duration of water storage in the previous two weeks, cleaning frequency of water storage container and home-based water treatment.

Sanitation factors that were observed by the data collectors were: type of sanitation facility (latrine type), presence of handwashing facility with water near latrine, presence of latrine pit hole cover and feces observed on the floor and/or around the latrine. Latrine distance from home (latrine proximity) was measured using GPS (geographical positioning system). Sanitation factors that were self-reported by study participants were: latrine having been cleaned or not in the past two weeks, latrine-sharing status, safe/unsafe child feces disposal, solid waste disposal and liquid waste discharge methods.

Hygiene variables that were observed by data collectors were: presence of handwashing facility within/around the latrine, handwashing facility with water and soap within/around the latrine. Handwashing practice at critical times was also computed based on self-report of handwashing with water and soap at the five critical times (before preparing food, before feeding a child, before eating, after defecation and after cleaning a child who had defecated). The hygiene variable that was measured using self-report was mother/caregiver knowing that germs were removed by handwashing with water and soap. Child feeding practices that were self-reported by the mothers/caregivers were: child age (in months) at which food was started, child food/drink utensil storage, child food/drink storage place and bottle feeding.

### Data collection tool and quality assurance

Data were collected using a pre-tested structured questionnaire and on-the-spot observation checklist. A structured questionnaire was prepared in English, translated to Amharic (local language) and then re-translated to English to keep consistency. The questionnaire was adapted from relevant literature, the 2016 EDHS [33], the 2006 WHO and UNICEF Household Survey [39] and other published papers [21, 22, 40]. The questionnaire elicited information on the socio-economic/demographic and WASH variables listed above.

Five data collectors and one supervisor who were environmental health professionals with BSc were recruited. Two days’ training was provided for the data collectors and supervisor on the objectives of the study, ethical issues, the content of the questionnaire, and approaches to be used during data collection. The questionnaire was pre-tested near Woldia Town (in Mersa Town) on a sample 10 % of the size of the study sample based on which some amendments were made to the instrument. The principal investigator supervised the data collection process with the supervisor and supported the data collectors. All these aforementioned steps were taken to ensure the quality of the data.

Inter-observer reliability was ensured by providing clear definitions of events to be recorded, by training data collectors, and by providing feedback about discrepancies during daily supervision. We re-interviewed 10 % of the study participants using a different interviewer to check reliability of the information entered by different interviewers. The qualifications of the data collectors and the training they received reduced the likelihood of interviewer bias. During administration of the survey, the collected data were checked for completeness daily by the principal investigator and supervisors.

### Data management and analysis

The data were entered into EpiData version 3.1 and exported to SPSS (Statistical Package for the Social Sciences) version 21.0 software for data cleaning and analysis. Descriptive analyses such as frequencies and cross-tabulations were carried out to examine the distribution of each individual variable. The prevalence of acute diarrhea was determined by dividing the number of acute diarrhea cases in the two weeks prior to the survey by the total number of children included in the study.

Economic status (wealth index) of each household was determined using principal component analysis after checking its assumptions for communality value > 0.5, KMO (sampling adequacy) > 0.5, which was 0.837 with *p*-value < 0.001 and complex structure factor (eigenvalue) greater than 1. The total variance explained by the final component was 67.42 %. Then, the wealth index was computed by calculating the mean and establishing five wealth categories relative to the mean: lowest, second, middle, fourth, and highest.

The presence of multi-collinearity among independent variables was checked using standard error at the cutoff value of 2; we found a maximum standard error of 1.68, indicating no multi-collinearity. None of the covariates were collinear (Pearson’s correlation coefficient *r* > 0.8). A binary logistic regression model was applied for data analysis. Bivariable analysis (COR [crude odds ratio]) was carried out to identify associations between the dependent variable and each independent variable. Variables with *p*-value < 0.2 from bivariable analysis were retained for subsequent multivariable analysis (AOR [adjusted odds ratio]) to control for possible confounders. Then, from the multivariable analysis, odds ratios with 95 % CI were calculated to measure the strength of the association. Level of statistical significance was declared at *p*-value ≤ 0.05. During the adjusted multivariable analysis, application of the Hosmer-Lemeshow goodness-of-fit test showed the model to be good (*p*-value = 0.765).

## Results

### Socio‐demographic and economic characteristics

Of the 485 children under five who were eligible, 484 were enrolled, for a response rate of 99.8 %. The mean age of mothers/caregivers was 35.94 (± 7.45 SD [standard deviation]) years. About one-fifth of the study participants (*n* = 105, 21.7 %) were 20–29 years of age and half (*n* = 241, 49.8 %) were 30–39 years. One-third of mothers/caregivers 156 (32.2 %) had no formal education and 212 (43.8 %) had attained a primary level education. Three-fourths of the households (*n* = 372, 76.9 %) were occupied by five or fewer persons; mean household size was 4.27 (± 1.2) persons. More than two-thirds 342 (70.7 %) of householders owned their own house, whereas about one-fifth 99 (20.5 %) of householders had a rented house. One-fifth of study participants’ wealth status was categorized as lowest (*n* = 97, 20.0 %), whereas about one-tenth of them were categorized as highest (*n* = 57, 11.8 %) (Table 1).

**Table 1:** Bivariable analysis of socio-demographic and economic factors with acute diarrhea among children under five in Woldia Town, northeast Ethiopia, March to June 2018

Variable	Frequency	Acute diarrhea		COR (95% CI)	***p***-value
		Yes	No		
	***n***(%)	***n***(%)	***n***(%)		
**Mother’s/caregiver’s age (in years)**					
20-29	105(21.7)	15(14.3)	90(85.7)	1	
30-39	241(49.8)	51(21.2)	190(78.8)	1.61(0.86-3.02)	0.137
40-49	112(23.1)	13(11.6)	99(88.4)	0.79(0.36-1.75)	0.557
≥50	26(5.4)	6(23.1)	20(76.9)	1.80(0.62-5.21)	0.279
**Ethnicity**					
Amhara	462(95.5)	79(17.1)	383(82.9)	0.55(0.21-1.45)	0.230
Other (Tigre, Agew and Oromo)	22(4.5)	6(27.3)	16(72.7)	1	
**Religion**					
Orthodox	335(69.2)	58(17.3)	277(82.7)	0.95(0.57-1.57)	0.829
Muslim or Protestant	149(30.8)	27(18.1)	122(81.9)	1	
**Mother’s/caregiver’s educational level**					
No formal education	156(32.2)	38(24.4)	11875.6)	3.33(1.33-8.30)	0.010
Primary	212(43.8)	34(16.0)	178(84.0)	1.97(0.79-4.93)	0.145
Secondary	48(9.9)	7(14.6)	41(85.4)	1.76(0.55-5.62)	0.337
College or above	68(14.1)	6(8.8)	62(91.2)	1	
**Mother’s/caregiver’s occupation**					
Day laborer	12(2.5)	6(50.0)	6(50.0)	5.68(1.74-18.55)	0.004
Farmer	39(8.1)	13(33.3)	26(66.7)	2.84(1.34-6.02)	0.007
Student	30(6.2)	6(20.0)	24(80.0)	1.42(0.54-3.71)	0.475
Government employee	53(10.9)	7(13.2)	46(86.8)	0.86(0.36-2.06)	0.741
Private employee	28(5.8)	6(21.4)	22(78.6)	1.55(0.58-4.08)	0.376
Merchant	75(15.5)	10(13.3)	65(86.7)	0.87(0.41-1.85)	0.724
Housewife	247(51.0)	37(15.0)	210(85.0)	1	
**Marital status**					
Married	365(75.4)	66(18.1)	299(81.9)	1.68(0.64-4.42)	0.296
Widowed	31(6.4)	8(25.8)	23(74.2)	2.64(0.77-9.06)	0.122
Divorced	45(9.3)	6(13.3)	39(86.7)	1.17(0.33-4.16)	0.809
Single	43(8.9)	5(11.6)	38(88.4)	1	
**Father’s occupation** (*N* = 365)					
Farmer	154(42.2)	29(18.8)	125(81.2)	1.88(0.84-4.19)	0.122
Day laborer	19(5.2)	8(42.1)	11(57.9)	5.89(1.87-18.52)	0.002
Government employee	72(19.7)	10(13.9)	62(86.1)	1.31(0.50-3.42)	0.584
Private employee	38(10.4)	10(26.3)	28(73.7)	2.89(1.07-7.87)	0.038
Merchant	82(22.5)	9(11.0)	73(89.0)	1	
**Father’s educational level** (*N* = 365)					
No formal education	49(13.4)	11(22.4)	38(77.6)	2.86(1.06-7.68)	0.037
Primary	199(54.5)	41(20.6)	158(79.4)	2.56(1.15-5.73)	0.022
Secondary	30(8.2)	6(20.0)	24(80.0)	2.47(0.78-7.82)	0.124
College or above	87(23.9)	8(9.2)	79(90.8)	1	
**Household size (persons)**					
>5	112(23.1)	26(23.2)	86(76.8)	1.60(0.95-2.70)	0.075
≤5	372(76.9)	59(15.9)	313(84.1)	1	
**House ownership**					
Own	342(70.7)	61(17.8)	281(82.2)	0.82(0.37-1.80)	0.621
Rent	99(20.4)	15(15.2)	84(84.8)	0.68(0.27-1.69)	0.400
Neither own nor rent	43(8.9)	9(20.9)	34(79.1)	1	
**Wealth index**					
Lowest	97(20.0)	26(26.8)	71(73.2)	2.62(1.05-6.50)	0.038
Second	152(31.4)	25(16.4)	127(83.6)	1.41(0.57-3.46)	0.458
Middle	104(21.5)	16(15.4)	88(84.6)	1.30(0.50-3.37)	0.591
Fourth	74(15.3)	11(14.9)	63(85.1)	1.25(0.45-3.45)	0.671
Highest	57(11.8)	7(12.3)	50(87.7)	1	

*1* reference category, *COR* crude odds ratio, *CI* confidence interval

### Index child‐related characteristics

The sexes of participating children were almost equal 248 (51.2 %) females and 236 (48.8 %) males. The mean age of participating children was 27.69 (± 16.4) months. More than half 264 (54.6 %) of the children were between 24 and 59 months old and almost one-fifth 112 (23.1 %) were 12–23 months. Almost all households (*n* = 466, 96.3 %) had only one child under five and nearly half (*n* = 236, 48.8 %) of the children were male (Table 2).

**Table 2:** Bivariable analysis of index child related factors with acute diarrhea among children under five in Woldia Town, northeast Ethiopia, March to June 2018

Variable	Frequency	Acute diarrhea		COR (95% CI)	***p***-value
		Yes	No		
	***n***(%)	***n***(%)	***n***(%)		
**Index child sex**					
Female	248(51.2)	46(18.5)	202(81.5)	1.15(0.72-1.84)	0.559
Male	236(48.8)	39(16.5)	197(83.5)	1	
**Number of children under five in the household**					
Two or more children	18(3.7)	5(27.8)	13(72.2)	1.86(0.64-5.35)	0.253
One child	466(96.3)	80(17.2)	386(82.8)	1	
**Index child age (months)**					
0-5	44(9.1)	10(22.7)	34(77.3)	1.70(0.48 -3.16)	0.673
6-11	64(13.2)	20(31.3)	44(68.7)	2.62(1.42-7.21)	0.003
12-23	112(23.1)	16(14.3)	96(85.7)	0.96(0.55-1.58)	0.485
24-59	264(54.6)	39(14.8)	225(85.2)	1	
**Birth order of index child**					
First	123(25.4)	19(15.4)	104(84.6)	0.65(0.30-1.37)	0.255
Second	122(25.2)	16(13.1)	106(86.9)	0.53(0.25-1.16)	0.113
Third	78(16.1)	19(24.4)	59(75.6)	1.14(0.53-2.46)	0.743
Fourth	93(19.2)	16(17.2)	77(82.8)	0.73(0.33-1.61)	0.441
Fifth or above	68(14.1)	15(22.1)	53(77.9)	1	

*1* reference category, *COR* crud odds ratio, *CI* confidence interval

### Water‐related characteristics

The main source of drinking water for almost two-thirds of the households was a private tap 307 (63.4 %), whereas for almost one-fifth 91 (18.8 %) of households it was a public tap. For more than three-fourths 403 (83.3 %) of households, the main source of water was within 30 minutes’ walk. About two-thirds 320 (66.1 %) of the households’ water consumption per capita per day was less than 20 l, with a mean of 15.48 (± 8.137) liters. Three-fourths 363 (75.0 %) of households had experienced water supply interruption from the main source during the two weeks prior to the survey. Less than half 217 (44.8 %) of households had stored water for 3 or more days during the two weeks prior to the survey. Only one-tenth 49 (10.1 %) of households practiced home-based water treatment. More than half 269 (55.6 %) of households always cleaned the water storage container before fetching water, whereas almost one-third 157 (32.4 %) cleaned the water storage container weekly (Table 3).

**Table 3:** Bivariable analysis of water-related factors with acute diarrhea in Woldia Town, northeast Ethiopia, March to June 2018

Variable	Frequency	Acute diarrhea		COR (95% CI)	***p***-value
	***n***(%)	Yes	No		
		***n***(%)	***n***(%)		
**Main source of drinking water**					
Public tap	91(18.8)	29(31.9)	62(68.1)	3.52(1.41-7.17)	0.001
Protected well	44(9.1)	11(25.0)	33(75.0)	2.51(1.17-5.40)	0.019
Protected spring	42(8.7)	9(21.4)	33(78.6)	2.05(0.91-4.62)	0.084
Private tap	307(63.4)	36(11.7)	271(88.3)	1	
**Walking time to obtain drinking water from main source**					
30 minutes or longer	81(16.7)	21(25.9)	60(74.1)	1.85(1.14-4.62)	0.030
Less than 30 minutes	403(83.3)	64(15.9)	339(84.1)	1	
**SDGs water supply safely managed criteria**					
**Quantity of water consumption per capita per day**					
<20 liters	320(66.1)	73(22.8)	247(77.2)	3.74(1.97-7.12)	<0.001
≥20 liters	164(33.9)	12(7.3)	152(92.7)	1	
**Water supply interruption during the past two weeks** **(reliability of water supply)**					
Yes	363(75.0)	68(18.7)	295(81.3)	1.41(0.79-2.51)	0.243
No	121(25.0)	17(14.1)	104(85.9)	1	
**Water source connection**					
In-house connection	98(20.2)	6(6.1)	92(93.9)	1	
Yard connection	207(42.8)	30(14.5)	177(85.5)	2.60(1.04-6.47)	0.040
Connection outside the compound	179(37.0)	49(27.4)	130(72.6)	5.77(2.05-14.07)	<0.001
** Bacteriological water quality and chemical contamination of main source of drinking water**^**a**^					
**Water storage duration during the past two weeks**					
≥3 days	217(44.8)	39(18.0)	178(82.0)	1.05(0.66-1.68)	0.831
<3 days	267(55.2)	46(17.2)	221(82.8)	1	
**Container used to store water**					
Plastic bucket with lid/pot with lid	256(52.9)	209(81.6)	47(18.4)	1.12(0.70-1.80)	0.625
Small-necked jerry cans with lid	228(47.1)	38(16.7)	190(83.3)	1	
**Frequency of cleaning of water storage container**					
Weekly	157(32.4)	23(14.6)	134(85.4)	0.73(0.43-1.26)	0.259
Once per two weeks	58(12.0)	11(19.0)	47(81.0)	1.00(0.49-2.06)	0.999
Always before fetching water	269(55.6)	51(19.0)	218(81.0)	1	
**Home-based water treatment**					
No	435(89.9)	79(18.2)	356(81.8)	1.59(0.65-3.87)	0.306
Yes	49(10.1)	6(12.2)	43(87.8)	1	

*1* reference category, *COR* crud odds ratio, *CI* confidence interval

^a^Our study did not consider the primary test of bacteriological water quality and chemical contamination. But, the secondary data report during the study period (March to June 2018) from the Woldia town water and sewerage office showed that the water quality met WHO guidelines of 0 cfu/100 ml for *E. coli* (*Escherichia* *coli*) indicator at the water source and also met the WHO guidelines for primary chemical status at the water source [41]

### Sanitation‐related characteristics

More than three-fourths 385 (79.5 %) households used an unimproved latrine and about one-fifth 99 (20.5 %) of households used an improved latrine. Less than half 212 (43.8 %) of households used a latrine shared with two or more households. During the two weeks prior to the survey, half 248 (51.2 %) of the households reported they had not cleaned the latrine and 236 (48.8 %) of households reported they had cleaned the latrine at least once. The proximity of the latrine to home for more than half 281 (58.1 %) of households was 15 m or more and for 203 (41.9 %) was less than 15 m. Feces were observed on the floor and/or around the latrine of more than three-fourths 381 (78.7 %) of the latrines. The majority of mothers/caregivers (*n* = 275, 56.8 %) practiced safe child feces disposal and 209 (43.2 %) practiced unsafe child feces disposal. Liquid waste was discharged improperly by two-thirds 316 (65.3 %) of the households and properly by 168 (34.7 %) of households (Table 4).

**Table 4:** Bivariable analysis of sanitation-related factors with acute diarrhea among under-five children in Woldia Town, northeast Ethiopia, March to June 2018

Variable	Frequency	Acute diarrhea		COR (95% CI)	***p***-value
		Yes	No		
	***n***(%)	***n***(%)	***n***(%)		
**Type of sanitation facility**^**b**^					
Unimproved	385(79.5)	80(20.8)	305(79.2)	4.93(1.94-9.5)	<0.001
Improved	99(20.5)	5(5.1)	94(94.9)	1	
**Sharing status of latrine**^**a**^					
Not shared	272(56.2)	40(14.7)	232(85.3)	0.64(0.40-1.02)	0.063
Shared	212(43.8)	45(21.2)	167(78.8)	1	
**Latrine cleaned in the past two weeks**					
Not cleaned	248(51.2)	52(21.0)	196(79.0)	1.63(1.01-2.63)	0.045
Cleaned at least once	236(48.8)	33(14.0)	203(86.0)	1	
**Proximity of latrine to home**					
<15 meters	203(41.9)	39(19.2)	164(80.8)	1.18(0.64-1.68)	0.364
≥15 meters	281(58.1)	47(16.7)	234(83.3)	1	
**Latrine pit hole had a cover**					
No	422(87.2)	78(18.5)	344(81.5)	1.78(0.76-3.50)	0.321
Yes	62(12.8)	7(11.3)	55(88.7)	1	
**Feces observed on the floor and/or around the latrine**					
Yes	103(21.3)	28(27.2)	75(72.8)	2.12(1.13-4.95)	0.003
No	381(78.7)	57(15.0)	324(85.0)	1	
**Child feces disposal method**					
Unsafe	209(43.2)	57(27.3)	152(72.7)	3.31(2.02-5.43)	<0.001
Safe	275(56.8)	28(10.2)	247(89.8)	1	
**Solid waste disposal method**					
Improper disposal	280(57.8)	47(16.8)	233(83.2)	0.88(0.55-1.41)	0.599
Proper disposal	204(42.2)	38(18.6)	166(81.4)	1	
**Liquid waste discharge method**					
Improper discharge	316(65.3)	46(14.6)	270(85.4)	0.56(0.26-1.25)	0.268
Proper discharge	168(34.7)	39(32.2)	129(76.8)	1	

*1* reference category, *COR* crud odds ratio, *CI* confidence interval

^a^Latrine sharing means one latrine used by two or more household members

^b^All households did not use improved sanitation facility, which means safely-managed sanitation was not implemented at all households

### Handwashing‐related characteristics

Handwashing facilities within/around the latrine were available in one-fifth 102 (21.1 %) of the latrines, of which more than three-fourths 80 (78.4 %) had no water and soap and only 22 (21.6 %) had water and soap. More than half 259(53.5 %) of the study participants reported poor handwashing practices at critical times and 225 (46.5%) had good handwashing practice at critical times. A majority 406 (83.9 %) of mothers/caregivers knew about germs being removed by handwashing with water and soap. A majority 430 (88.8 %) of the mothers/caregivers had information about diarrhea being prevented by handwashing with water and soap (Table 5).

**Table 5:** Bivariable analysis of hygiene-related factors with acute diarrhea among under-five children in Woldia Town, northeast Ethiopia, March to June 2018

Variable	Frequency	Acute diarrhea		COR (95% CI)	***p***-value
		Yes	No		
	***n***(%)	***n***(%)	***n***(%)		
**Handwashing facility within/around the latrine**					
No	382(78.9)	74(19.4)	308(80.6)	1.64(1.14-5.32)	0.041
Yes	102(21.1)	13(12.7)	89(87.3)	1	
**Handwashing facility with water and soap within/near latrine** **(*n*= 102)**					
No	80(78.4)	10(12.5)	70(87.5)	0.90 (0.43-1.32)	0.456
Yes	22(21.6)	3(13.6)	19(86.4)	1	
**Handwashing practice at critical times**					
Poor practice	259(53.5)	60(23.2)	199(76.8)	2.41(1.45-4.00)	0.001
Good practice	225(46.5)	25(11.1)	200(88.9)	1	
**Mother/caregiver knew about germs being removed by handwashing with water and soap**					
No	78(16.1)	13(16.7)	65(83.3)	0.93(0.49-1.77)	0.821
Yes	406(83.9)	72(17.7)	334(82.3)	1	
**Mother/caregiver had information about diarrhea being prevented by handwashing with water and soap**					
Had no information	54(11.2)	20(37.0)	34(70.0)	3.30(1.79-6.09)	<0.001
Had information	430(88.8)	65(15.1)	365(84.9)	1	
**Mother/caregiver had information about WASH**					
Had no information	47(9.7)	16(34.0)	31(70.0)	2.75(1.43-5.30)	0.002
Had information	437(90.3)	69(15.8)	368(84.2)	1	

*1* reference category, *COR* crud odds ratio, *CI* confidence interval

### Child feeding practices

Of the 484 children under five, 462 (95.5 %) consumed food other than breast milk and only 22 (4.5 %) were on breast milk feedings only. Almost half 254 (52.5 %) of the children had started complementary food before six months of age and 208 (43.0 %) began complementary food after six months of age. Almost one-third 147 (31.8 %) of mothers/caregivers stored their children’s food/drink on a covered shelf, but 234 (50.7 %) stored it on an open shelf. Almost one-fifth 86 (17.7 %) of children were fed by bottle (Table 6).

**Table 6:** Bivariable analysis of child feeding related factors with acute diarrhea among under-five children in Woldia Town, northeast Ethiopia, March to June 2018

Variable	Frequency	Acute diarrhea		COR (95% CI)	***p***-value
		Yes	No		
	n(%)	n(%)	n(%)		
**Child consumed food other than breast milk**					
Yes	462(95.5)	79(17.1)	383(82.9)	0.55(0.21-1.45)	0.227
No	22(4.5)	6(27.3)	16(72.7)	1	
**Child food start age in months**					
Less than 6 months	254(52.5)	45(17.7)	209(82.3)	0.57(0.21-1.55)	0.574
6 month or above	208(43.0)	34(16.3)	174(83.7)	0.52(0.19-1.43)	0.205
Child <6 months and breastfed only	22(4.5)	6(27.3)	16(72.7)	1	
**Child food/drink utensil storage (*N*** **= 462)**					
Anywhere by covering	30(6.5)	9(30.0)	21(70.0)	1.90(0.82-4.45)	0.137
Anywhere by not covering	51(11.0)	18(35.3)	33(64.7)	2.42(1.25-4.70)	0.009
With covered utensils on shelf	147(31.8)	9(6.1)	138(93.9)	0.29(0.14-0.61)	0.001
With uncovered utensils on shelf	234(50.7)	43(18.4)	191(81.6)	1	
**Child food/drink storage (*N*** **= 462)**					
Refrigerator	175(37.9)	11(6.3)	164(93.7)	1	
Open shelf	150(32.4)	35(23.3)	115(76.7)	4.54(2.21-9.31)	<0.001
Covered shelf	84(18.2)	9(10.7)	75(89.3)	1.79(0.71-4.50)	0.216
Anywhere	53(11.5)	24(45.3)	29(54.7)	12.34(5.46-27.98)	<0.001
**Child bottle fed**					
Yes	86(17.8)	19(22.1)	67(77.9)	1.43(0.80-2.53)	0.225
No	398(82.2)	66(16.6)	332(83.4)	1	

*1* reference category, *COR* crud odds ratio, *CI* confidence interval

### Prevalence and WASH associated determinants of acute diarrhea

The overall two-week period prevalence of acute diarrhea among the children was 17.6 % (95 % CI: 14.2–21.0 %). Children from households with water consumption of less than 20 l per capita per day were 2.45 times more likely to develop acute diarrhea than children from households in which water consumption was ≥ 20 l per capita per day (AOR = 2.45; 95 % CI: 1.36–5.84). Under-five children from households with an unimproved sanitation facility were 3.57 times more likely to develop acute diarrhea than children from households that used an improved sanitation facility (AOR = 3.57, 95 % CI: 1.64–6.51) (Table 7).

On the other hand, the odds of having acute diarrhea among children of mothers/caregivers with poor handwashing practice at critical times were 1.85 times as high as those whose mothers/caregivers practiced good handwashing at critical times (AOR = 1.85; 95 % CI: 1.34–3.56). Similarly, the odds of acute diarrhea among children whose mothers/caregivers practiced unsafe child feces disposal were 2.51 times as high as those whose mothers/caregivers practiced safe child feces disposal (AOR = 2.51; 95 % CI: 1.69–4.64). The odds of acute diarrhea among children whose mothers/caregivers had no information about acute diarrhea being prevented by handwashing with water and soap were 3.12 times higher than for those children whose mothers/caregivers who had information about acute diarrhea being prevented by handwashing with water and soap (AOR = 3.12; 95 % CI: 1.64–6.27) (Table 7).

**Table 7:** Water, sanitation and hygiene- related factors associated with acute diarrhea among under-five children from multivariable logistic regression analysis* in Woldia Town, northeast Ethiopia, March to June 2018

Variable	AOR (95% CI)	***p***-value
**Quantity of water consumption per capita per day**		
<20 liters	2.45(1.36-5.84)	<0.001
≥20 liters	1	
**Type of sanitation facility**		
Unimproved	3.57(1.64-6.51)	<0.001
Improved	1	
**Handwashing practice at critical times**		
Poor practice	1.85(1.34-3.56)	0.001
Good practice	1	
**Child feces disposal**		
Unsafe	2.51(1.69-4.64)	0.001
Safe	1	
**Mother/caregiver had information about acute diarrhea being prevented by handwashing with water and soap**		
Had no information	3.12(1.64-6.27)	<0.001
Had information	1	

*1* reference category, *AOR* adjusted odds ratio, *CI* confidence interval

*Variables that had a *p-*value < 0.2 from the bivariate analysis were included into the multivariable logistic regression model. A total of 25 variables that were adjusted/controlled for multivariable analysis included mother’s/caregiver’s age, mother’s/caregiver’s educational level, mother’s/caregiver’s occupation, marital status, father’s occupation, father’s educational level, wealth index, index child age, birth order of index child, main source of drinking water, time to obtain drinking water, quantity of water consumption per capita per day, type of sanitation facility, sharing status of latrine, latrine cleaning in the past two weeks, feces observed on the floor and/or around the latrine, child food/drink utensil storage, child food/drink storage, mother/caregiver knew about germs being removed by handwashing with water and soap, handwashing facility within/around the latrine, handwashing practice at critical times, mother/caregiver information about WASH, mother/caregiver information about acute diarrhea being prevented by handwashing with water and soap and child feces disposal method

## Discussion

This study examined the prevalence and WASH-associated factors of acute diarrhea among children under five in Woldia Town, Ethiopia. The findings revealed that the prevalence of acute diarrhea among children under five in the two-week study period was 17.6 % (95 % CI: 14.2–21.0 %). After adjustment for potential confounders, acute diarrhea was found to be significantly associated with water consumption per capita per day (< 20 l), used unimproved sanitation facility, mothers/caregivers practicing poor handwashing at critical times, mothers/caregivers practicing unsafe child feces disposal methods and mothers/caregivers had no information about acute diarrhea being prevented by handwashing with water and soap.

The prevalence of acute diarrhea in Woldia Town was lower than found in studies conducted in Uganda (32.0 and 24.1 %) [42, 43], Senegal (26.0 %) [44], rural Burundi (32.6 %) [45], and in different areas of Ethiopia (30.6 %, 28.9 %, 30.5 %, 22.1 %) [17, 20, 46, 47]. The lower prevalence of acute diarrhea in our study might be due to the implementation of health extension programs by the Ethiopian government. The lower prevalence might be due also to different data collection seasons and differences in important factors such as the provision of basic sanitation services.

Diarrhea prevalence in Woldia was similar to that found in Farta District (18.0 % diarrhea prevalence) [48], and in towns included in the 2005 EDHS (18 %) [49]. However, prevalence in our study was higher than that found in the 2016 EDHS (12.2 %) [50] and in other studies conducted in different parts of Ethiopia, such as Wolayta Soddo Town (11 %) [11], Awi and West and East Gojjam zones (13.5 %) [51], Debre Berhan Town (12.2 %) [52], and Jigjiga Town (14.6 %) [53]. The higher prevalence in the current study may be due to differences in socio-demographic and WASH characteristics as well as differences in sample size and study periods.

In our study, children from households consuming less than 20 l of water per capita per day were 2.45 times more likely to develop acute diarrhea than children from households using 20 l or more per capita per day. The low consumption of water might be due to intermittent water supply, which is a fcator to acute diarrea as indicated elsewhere [22]. This findings agrees with those from two other rural areas in Ethiopia [54, 55]. This low water consumption might exposed for lower personal hygiene levels, which in turn can be a factor for acute diarreha among under-five children.

Our results also showed that the odds of acute diarrhea among under-five children were significantly higher for those whose mothers/caregivers had no information about acute diarrhea being prevented by handwashing with water and soap. A child whose mother/caregiver had no information about acute diarrhea being prevented by handwashing with water and soap was 3.12 times more likely to develop acute diarrhea than a child whose mother/caregiver had this information. This might be due to the fact that mothers/caregivers who get information about acute diarrhea being prevented by handwashing with water and soap from the news media and health professionals know more about the methods for prevention and management of diarrhea. These findings are consistent with other study in Ethiopia [56] and Iran [57]. Even though those studies dealt with diarrhea management and were not comparable with studies in other Ethiopian communities [54, 55], where under-fives in households lacking radios were more likely to develop acute diarrhea.

The odds of acute diarrhea among children whose mothers/caregivers had poor handwashing practices at critical times were 1.85 times as high as among those whose mothers/caregivers practiced good handwashing at critical times. Not washing hands at critical times exposes people to microorganisms because hands are the most exposed body parts after high-risk activities such as defecation and cleaning a child who has defecated; after this, handwashing is essential before preparing food, before feeding a child, and before eating [58]. The outcome of this study about the association between poor handwashing practice at critical times and the occurrence of acute diarrhea among children under five is in line with the results of other studies in Zambia [59], and Nigeria [60] but not inline with studies in two other Ethiopian communities [54, 55].

Acute diarrhea among children under five was also associated with mothers’/caregivers’ use of unsafe child feces disposal methods. The odds of children whose mothers/caregivers practiced unsafe child feces disposal having acute diarrhea were 2.51 times as high as children whose mothers/caregivers practiced safe child feces disposal. Children’s stools tend to carry a higher pathogen load than those of adults, and many children play in contaminated areas; safe feces disposal is therefore critical for reducing the number of diarrhea cases [61]. This finding is in agreement with studies in three other Ethiopian communities [54–56] and in Indonesia [62] but not with a study Farta districts in central Ethiopia [48] and in Iraq [63], Sudan [64], and rural Burundi [45].

Although we did not study mode of service for water supply using SGDs criteria for safely managed water supply, our findings indicated that a majority of the households received less than 20 l/c/d water supply even if they had access to water at a yard or in-house connection. This indicated a problem with the reliability of water supply in Woldia Town, which might be due to frequent interruption and or low amount of drinking water production as the source. If people are getting less water below the SGDs standared of daily water consumption per capita per day where there is a yard or in-house connection, the issue is due to the reliability of supply, which is one of the criteria for SDGs safely managed system. Therefore, intervention is needed to improve the reliability of the water supply system in Woldia town.

Our study did not consider the primary test of water quality for bacteriological (*Escherichia coli* [*E. coli*] indicator and primary chemical contamination test. But, the secondary data report during the study period (March to June 2018) from the Woldia Town water and sewerage office showed that the water quality meet WHO guidelines of 0 cfu/100 ml for *E. coli* indicator at the water source and also meet the WHO guidelines for primary chemical status at the water source [41].

Our findings also indicted more than three-fourth (79.5 %) of households used unimproved sanitation, which means safely managed sanitation was not implemented in the majority of the households. This may have contributed to the high prevalence of acute diarrhea in our study area. To meet the SDGs safely managed sanitation, households should use improved sanitation facilites for excreta to be safely disposed in situ and then excreta should be transported and treated offsite.

### Limitations of the study and gaps for further research

The measurement of the acute diarrhea and some of the WASH factors were self-reported, which may have been affected by social desirability bias, where over-reporting and/or under-reporting might have happened [65]. Since our study also did not consider the Rota vaccination, deworming, measles and vitamin-A supplementation as factors, we recommend future researchers consider them to find more holistic evidence. In addition, this study was unable to examine seasonal differences in diarrhea occurrence. Since previous studies have showed that diarrhea incidence tends to peak during the rainy season [66], further studies are encouraged to be conducted by considering the impact of seasonal variation on diarrhea.

Our study did not consider the hierarchy of water supply services–in-house connection, yard connection, outside-the-compound connection. Further studies are highly encouraged to consider the implications of these various services as related to the household water demands, including consideration of a private or shared yard connection, the number of households using a shared connection, and evaluation of the water demand of the households. Furthermore, future studies should evaluate how well the water supply system meets safely-managed definitions of SDGs, including reliability, quality, and quantity of supply (at premises/yard, about 50 l/c/day and in-house connection, about 80 l/c/day).

Therefore, further studies are highly encouraged to investigate the bacteriological contamination of water from source to point-of-use to control acute diarrhea among under-five children. Last, since this study was conducted in Woldia Town only, and findings cannot be generalized to the national level, a large-scale study that addresses a national context is highly recommended.

## Conclusions

The study revealed a relatively high two-week prevalence of acute diarrhea (17.6 %) among children under five. The study identified water consumption of less than 20 liters per capita per day, use of unimproved sanitation facility, mothers’/caregivers’ poor handwashing practice at critical times, unsafe child feces disposal methods and mother/caregiver having no information about acute diarrhea being prevented by handwashing with water and soap as significantly associated with acute diarrhea. Therefore, government organizations and stakeholders should strengthen urban WASH programs in Woldia, focusing on the factors identified in this study. Strengthening the urban WASH program may contribute to achieving the SDGs 2030, mainly Goal 3 about health and wellbeing and Goal 6 about water supply and sanitation, but also Goal 11 about creating inclusive and sustainable cities. To achieve these goals, there is an urgent need to prioritize health education focusing on the software (behavior) component of handwashing with water and soap at critical times and safe disposal of child feces. Furthermore, focusing on the hardware component of providing improved sanitation facilities and sufficient safe water supply with sufficient water consumption per capita per person are essential.

## Data Availability

The dataset that was used during the current study is available from the corresponding author on reasonable request.

## Abbreviations

CI: Confidence interval
COR: Crude odds ratio
SDGs: Sustainable Development Goals
UNICEF: United Nations International Children’s Emergency Fund
WHO: World Health Organization
EDHS: Ethiopian Demographic and Health Survey
WASH: Water, sanitation and hygiene
